# Effect of experimental soil disturbance and recovery on structure and function of soil community: a metagenomic and metagenetic approach

**DOI:** 10.1038/s41598-017-02262-6

**Published:** 2017-05-23

**Authors:** Soobeom Choi, Hokyung Song, Binu M. Tripathi, Dorsaf Kerfahi, Hyoki Kim, Jonathan M. Adams

**Affiliations:** 1 Department of Biological Sciences, College of Natural Sciences, Seoul National University, Gwanak-Gu, Seoul 08826 Republic of Korea; 2 Arctic Research Center, Korea Polar Research Institute, Incheon-si, Gyeonggi-do 21990 Republic of Korea; 3 Celemics Inc., 371-17, Gasan-dong, Geumcheongu, Seoul 153-718 Republic of Korea

## Abstract

There has been little study of effects of disturbance on soil biota combining closely controlled experimental conditions and DNA-based methods. We sampled pots of soil at varying times following an initial simulated mass mortality event. Soil DNA was extracted at intervals up to 24 weeks after the event, and shotgun metagenomes sequenced using NextSeq. Compared to initial conditions, we found: consistent, sequential changes in functional metagenome and community structure over time, indicating successional niche differentiation amongst soil biota. As predicted, early successional systems had greater abundance of genes associated with motility, but fewer genes relating to DNA/RNA/protein metabolism, cell division and cell cycle. Contrary to predictions, there were no significant differences in cell signaling, virulence and defense-related genes. Also, stress related genes were less abundant in later succession. The early successional system had lower taxonomic diversity but higher functional gene diversity. Over time, community characteristics changed progressively, but by the end of the experiment had not returned to the ‘original’ state of the system before disturbance. Results indicated a predictable sequence of gene functions and taxa following disturbance, analogous to ecosystem succession for large organisms. It is unclear if and when the system would return to its pre-disturbance state.

## Introduction

Disturbance is widely regarded as an important influence on community structure of macro-organisms (those large enough to be seen with the naked eye). This is especially so for physical disturbances^1^, such as: forest fire, landslide, flood, storm damage, trampling of pasture by large herbivores, mowing and ploughing, involving the death of a large proportion of a community in a short period of time. For instance, the effects of such disturbances have been described for forest trees^2^, herbaceous plants^3^, algae^4^ and also corals^5^, amongst others. Physical disturbance of ecosystems releases nutrients by breakdown of organisms, making resources available and altering both the absolute and relative rates of ecosystem processes (e.g. primary productivity, secondary consumption, decomposition, nitrogen fixation), and qualitatively altering the main pathways involved in energy and nutrient fluxes^6–8^.

As well as changes in ecosystem processes, disturbance causes changes in community structure. After disturbance, ‘lottery effects’ provide ready access to resources for organisms in similar niches, regardless of their effectiveness in steady competition^9, 10^. By allowing exploitation of random opportunities, it may be possible for more species to coexist in a disturbed community^11^. However, while disturbance may increase diversity in communities with moderate levels of disturbance, beyond a certain point more frequent disturbance causes a decline in diversity^5^. By creating a range of niches for differing degrees of dispersal ability, disturbance is often important in adding to community diversity of macro-organisms^9, 12^.

To investigate the effect of disturbance on diversity, community structure and ecosystem functioning, studies have been conducted in both naturally or artificially disturbed systems^10, 13, 14^. However, in contrast to studies on larger organisms, the responses of microbial communities to disturbance and primary successional environments has not been studied in terms of detailed community composition – and most of what has been done has tended to focus on observation of naturally occurring processes rather than experimental manipulation of controlled conditions^15–21^. As in the study of microbial ecology in general, culture-independent methods have provided new perspectives on the responses of microbial communities to disturbance, through both natural and anthropogenic processes^22–24^.

Several studies in bacterial ecology have concentrated on newly created or drastically disturbed habitats, such as the infant gut^25^, and rock pools^18^ explaining the mechanisms of assembly and reassembly during the colonization. In earlier studies using an experimental soil system, we^24^ studied the response of the assembly pattern of soil bacterial communities to repeated disturbance on a fine scale in a microcosm in incubated pots of soil. That study found distinct, predictable bacterial assemblages occurring at each recovery time step after disturbance, highlighting the potential role of niche differentiation of bacteria in relation to disturbance events, and its possible importance in producing the diversity of bacterial communities seen in nature. In the typical ecological view of disturbance (as used by Grime^9^ and Huston^26^) it is both the physical event and the deaths/injuries that occur immediately as a result of it. Hence, the event and its short term biological impact cannot be separated. The aim of our experiment here was to simulate in a general way the complete event ‘physical disturbance plus population crash’ followed by recolonization, as this is what has ecological meaning.

Here, we used a similar system to that used by Kim *et al*.^24^ but based instead upon a one-time nonspecific mass mortality events of soil biota, in small pots of soil kept in a laboratory growth chamber. Our aim in this project was to investigate the sequential changes which occur in community composition and functional genetics after a severe disturbance event affects a soil. In doing so, we hoped to add to understanding of 1) the important functional traits of soil organisms that are involved in community/ecosystem repair and resilience, 2) The impact of disturbance on taxonomic and gene function diversity and its rate of recolonization/recovery, 3) The degree to which different sets of soil organisms are specialized in terms of niches to exploit different stages of the recovery after disturbance, and 4) the extent to which the soil community and its function can return to its original state over several months.

## Our work was structured around several main questions

### What are the functional characteristics of early successional vs later successional soil communities?

The experiment explored changes in communities over a 168-day (24 week) time period. This, we propose, could be broadly analogous to the process of ecological succession seen elsewhere in nature for larger organisms, for example following a forest fire or other disturbance event. Since many microbes have potential doubling times of the order of hours to weeks depending on the species^27–31^, our 168-day experiment can be regarded as analogous to the studied time spans of secondary succession for plants or corals for example, where generation times vary from months to decades and successional systems span years to centuries^5, 32–34^. While we were unsure what span of time successional changes in the recovering soil community might require to be completed, our 24 week study was intended as a ‘first’ glimpse on a time scale that might plausibly show significant changes. This time scale is also relevant to many agricultural systems, which are ploughed or sprayed on a cycle of 6 months or so^35, 36^.

In the context of the broader background of community and ecosystem ecology for larger organisms, we made the following predictions for differences in metagenome functions for ‘earlier’ and ‘later’ successional bacterial communities. Many of these predicted traits can be seen in terms of the ‘r’ versus ‘K’ dichotomy in ecology^37^, although here it applies to aggregate traits of the whole community, rather than focusing on individual species.

### Cell division related genes will be more abundant in the early successional stage

Cell division is an aspect of growth, particularly in the prokaryotic world. Typically in ecology of larger organisms, in the early successional stages with abundant space and release of nutrients from dead organisms^38–40^, species grow fast and give many offspring, rapidly expanding their biomass^6, 41, 42^. Thus we expected this pattern to hold true in soil microbiota succession.

### At the later successional time stage, ‘housekeeping’ genes associated with basic metabolic functions will become relatively less common than genes for other extra functions associated with nutrient acquisition and competition

In secondary successional systems involving large organisms, mass death of organisms is accompanied by a release of nutrients which then become readily available for uptake^6^. It is less clear that natural disturbance of soil systems is always associated with mass death of soil biota – for example, entering dormancy might also be more important. However, in at least some situations such as sterilization of soil by heat from fire, mass death of soil biota is likely to predominate. In such a situation involving death of most soil biota, a similar initial increase in nutrient availability may be expected. As living biomass then increases, competition increases and usage of recalcitrant nutrients plus interference competition becomes more important^6^. We predicted that later successional stages in our soil systems would select for organisms carrying genes associated with sequestering nutrients from the more recalcitrant amongst the polymers associated with dead cells and soil humus, and also more genes associated with antagonism. In the classical ecology of large organisms, traits associated with nutrient acquisition and with interference competition are seen as being more abundant in later successional stages^6^.

### Genes related to cell-cell interactions will be more abundant in the later successional stages

It is generally agreed in ecology that later successional ecosystems have more intense and species-specific mutualistic and antagonistic interactions^6^. One example from forest successions would be the heavier reliance of plants on Ectomycorrhizal (EcM) fungi. We predicted similar trends towards intensity of (either positive or negative) organismic interactions in the late successional stage in the soil bacterial systems we were studying. We predicted that genes associated with antibiotic resistance, production of secondary compounds, quorum sensing and cell-cell recognition would become more important.

### Viruses and anti-virus defenses will become more abundant at later stages in the succession

High mortality of natural populations from diseases is associated with stable and high density host populations^43–45^. We predicted that in the early stages of succession after disturbance, a low total abundance of cells of all kinds should result in lower transmissivity and lower infection rates by viruses. As soil living biota build up in biomass, cell-cell neighbor distances should decrease (for all types of organism), resulting in greater transmission and infection rates. Likewise, strains of bacteria and archaea bearing CRISPR elements as anti-viral defenses^46^ will be more strongly selected in later stages, resulting in greater frequency of these in the metagenome.

### Motility related genes will be more abundant in early succession

In early successional organisms, dispersal is seen as a key trait in exploiting the environment fully by finding open space and resources to increase population^47, 48^. Following the initial disturbance event in this experiment, which involved autoclaving most of the soil (see Methods), we added back in by mixing a portion of the original ‘living’ soil, to serve as a source for recolonization of biota. In this situation, each particle of the added soil containing living organisms will be surrounded by a large proportion of potentially colonizable soil rich in resources from killed biota. This should select for organisms with the means to disperse within soil (e.g. flagellae), and the greater abundance of this trait should show up as genes for such characteristics as flagellae.

### Stress and dormancy genes will become more abundant in later succession

In secondary succession of larger organisms, resources are at first abundant, but become less abundant over time as they are sequestered in living biomass^49–51^. In late succession, not only nutrient shortage but interference competition between organisms becomes more common^41^. We predicted that likewise, there would be an increase in stress response genes related to both nutrient shortage and interference competition (e.g. antibiotic effects). We also predicted that dormancy genes would become more common, through selection of organisms able to survive in dormant form under nutrient shortage or interference competition.

## Results

From the 23 pot soil DNA samples in this study - including 1 original garden soil sample, 19 samples incubated after disturbance, and 3 non-disturbance incubation samples - 71,263,671 metagenome sequences were generated. Gene functional profiles were generated with the SEED Subsystems database of MG-RAST by using BLASTX. Phylogenetic information was extracted from the metagenomes using M5NR data bases using BLASTX. Information of samples and results of soil chemical analysis are shown in Supplementary Table S1 and Supplementary Table S2. A total of 27–42% of the sequences were annotated as protein using E < 1 × 10^5^ and 15-bp minimum alignment length on MG-RAST server. Also, from the same 23 pot soil DNA samples, 693,659 sequences of bacterial 16 S rRNA amplicon were generated. Subsampling at a level of 6,781 reads, we found 11,720 OTUs at the 97% similarity level.

### Dominant microbial taxa

**Figure 1:**
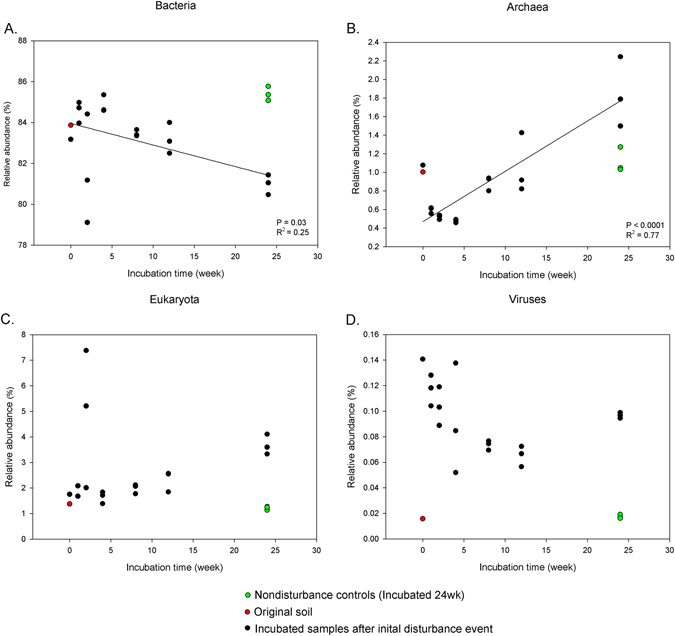
Relative abundance of each domain level assigned based on the M5NR database. Solid line represents linear regression fits to data. Relative abundance of (**A**) Bacteria, (**B**) Archaea, (**C**) Eukaryota, (**D**) Viruses. Colored points were not included in the statistics.

The majority of the metagenomic sequences among disturbance incubation samples were dominated by Bacteria (83.1% on average) followed by Eukaryota (2.7%), Archaea (0.9%) and Viruses (0.1%) according to the M5NR database.13.2% of reads were unassigned. Bacteria and Archaea relative abundances a showed linear inverse relationship to one another over time, with relative abundance of Bacteria decreasing but Archaea increasing (Fig. 1A,B). The relative abundance (%) of bacterial phyla observed in the metagenome data bacterial 16S rRNA amplicon sequences were shown in Supplementary Tables S3 and S4.

### Community composition in terms of functional genes and taxonomy

We generated nonmetric multidimensional scaling (NMDS) plots for summarizing taxonomic and functional information.

**Figure 2:**
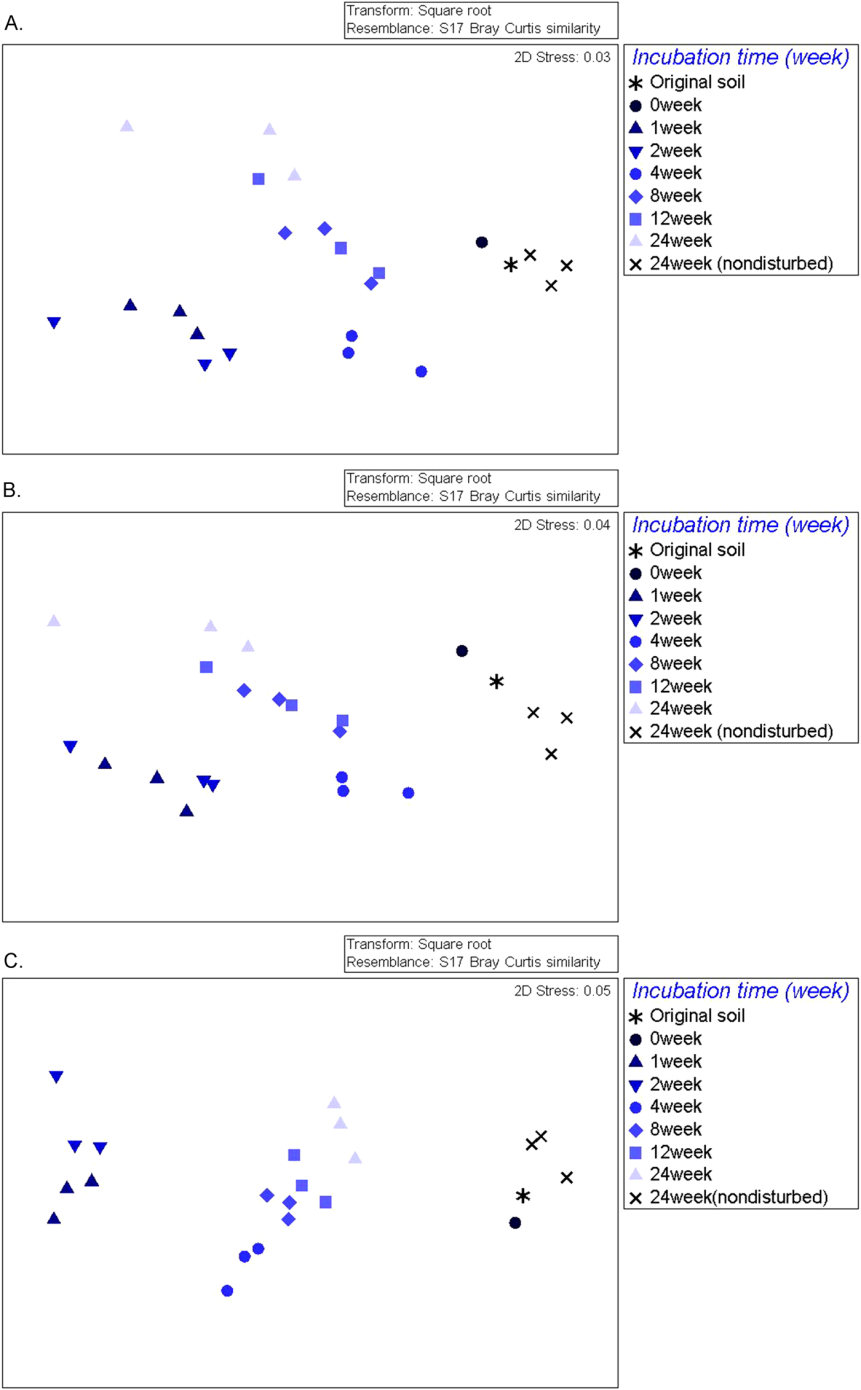
NMDS plot generated using weighted pairwise Unifrac distances between samples. (**A**) NMDS plot of shotgun metagenomic sequences based on M5NR taxonomic profile at family level, (**B**) NMDS plot of shotgun metagenomic sequences based on SEED functional profile at subsystem level 3, (**C**) NMDS plot of bacterial OTUs.

In terms of community taxonomic composition from the metagenome, the NMDS plot showed that replicates clearly clustered by time of incubation, in terms of their taxonomic composition (Fig. 2). With NMDS plots of taxonomic composition, we tested the mean of ranked dissimilarities between groups, using ANOSIM. Figure 2A,B and C shows R values of 0.793, 0.752 and 0.893 respectively (P < 0.001 in all statistics). Since the R values from ASNOSIM showed the groups are dissimilar in Fig. 2, the differing incubation time since disturbance contributed as a main factor in the distribution of the NMDS.

In Fig. 2A, the plot was generated at family level based on M5NR taxonomic profile. Nondisturbed control samples, also sieved but not partially sterilized and stored alongside the other pots at 25 °C for the 24 week period, clustered nearby the original soil in both taxonomic (Fig. 2A) and functional gene (Fig. 2B) terms. Of the experimental treatments which had gone through the disturbance event (90% of volume sterilized, followed by mixing back in 10% unsterilized soil), the earliest samples after the disturbance were most distinct from the original soil (and the controls). Over time, they moved across the NMDS plot, but after the 4 week stage the rate of change (as judged by the NMDS) the rate of change seems to slow, at least when judged subjectively by eye.

A very similar pattern was also observed for the functional gene composition of the soil biota, based on the metagenomes. In terms of functional level 3, based on the SEED functional profile, the earliest samples after the disturbance event likewise clustered furthest from original soil on the NMDS, then moved progressively across the NMDS plot (Fig. 2B). By the 4-week stage, this movement in terms of NMDS appeared to slow. Changes of functional gene profile over time thus showed a very similar trend to the taxonomic community changes.

The taxonomic structure of the bacterial community based on 16S rRNA amplicon sequencing showed a similar trend. At the OTU level, early stage (weeks 1, 2) bacterial communities were clustered furthest from original soil, and on the NMDS space shifted over time across the plot although in later stages there was no obvious movement towards the original and control soil (Fig. 2C).

We performed an Envfit analysis to discern the most important environmental factors in influencing the NMDS using R. The NMDS plot and Envfit of shotgun metagenomic sequences based on M5NR taxonomic profile at family level were the most strongly affected by TOC (P = 0.036, R² = 0.437, Supplementary Fig. S1A). Community composition in terms of functional genes level 3 of all biota based on M5NR taxonomic profile (Supplementary Fig. S1B) were influenced by TOC (P = 0.041, R² = 0.358). Community composition based on 16S rRNA amplicon data from bacterial OTUs was influenced by pH (P = 0.011, R² = 0.549) and TN (P = 0.023, R² = 0.455) (Supplementary Fig. S1C).

### Alpha diversity

**Figure 3:**
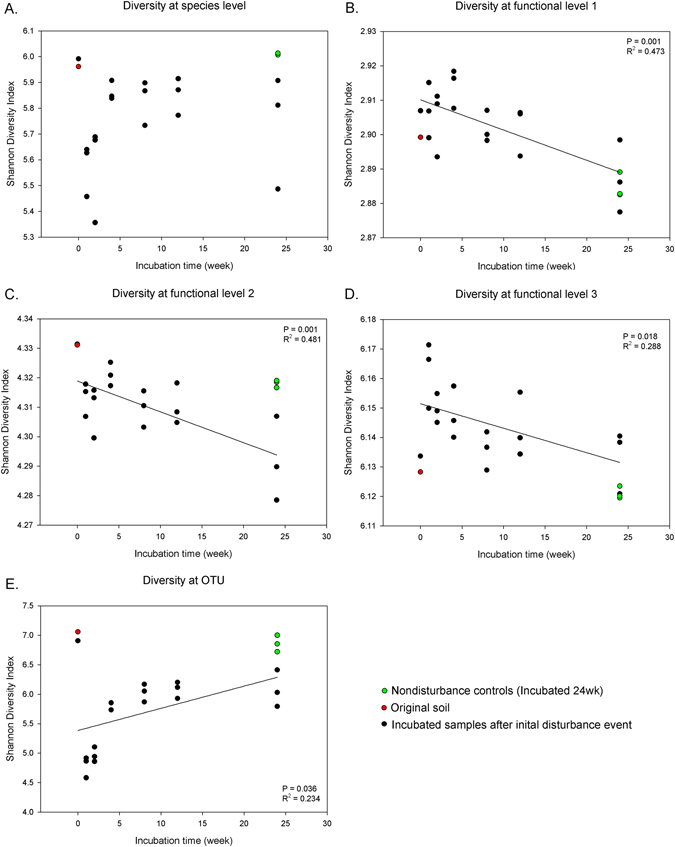
Taxonomic and Functional diversity (SEED database) at soil microbial communities by incubation time based on Shannon diversity index. Solid line represents linear regression fits to data. Diversity at (**A**) species level from all kingdoms (M5NR database), (**B**) functional level 1, (**C**) functional level 2, (**D**) functional level 3. (**E**) bacterial OTU level. Colored points were not included in the statistics.

We calculated diversity for the functional and taxonomic (16S rRNA amplicon and metagenome based) datasets based on the Shannon diversity index. Shannon diversity index was calculated based on M5NR taxonomy at species level as an indicator of diversity based on the species level, and SEED subsystem function at level 1, 2 and 3 by using R. In terms of alpha-diversity, the species level diversity (for taxonomically described published species) detected in the metagenomes was not affected by incubation time (P > 0.05, Fig. 3A). However, total functional gene Shannon diversity index, calculated for SEED subsystems level 1, 2 and 3, showed a significant declining trend of diversity over time (P < 0.05, Fig. 3B,C and D). For the 16S rRNA amplicon data, diversity at bacterial OTU level showed that Shannon diversity index increased over time since disturbance (P < 0.05, R² = 0.234, Fig. 3C), although its pattern was similar to diversity at species level from metagenome data (Fig. 3). The results of Mantel test showed that there was no correlation (P > 0.05) between taxonomic diversity and diversity of functional genes at levels 1, 2 or 3.

### Changes in functional gene categories with time after disturbance

To examine the effect of time succession after the experimental disturbance event on soil microbial functions, the functional profile of shotgun metagenomic sequences was analysed using the SEED database, and the metagenomic sequences were distributed into 28 functional gene categories (Level 1 SEED subsystems).

**Figure 4:**
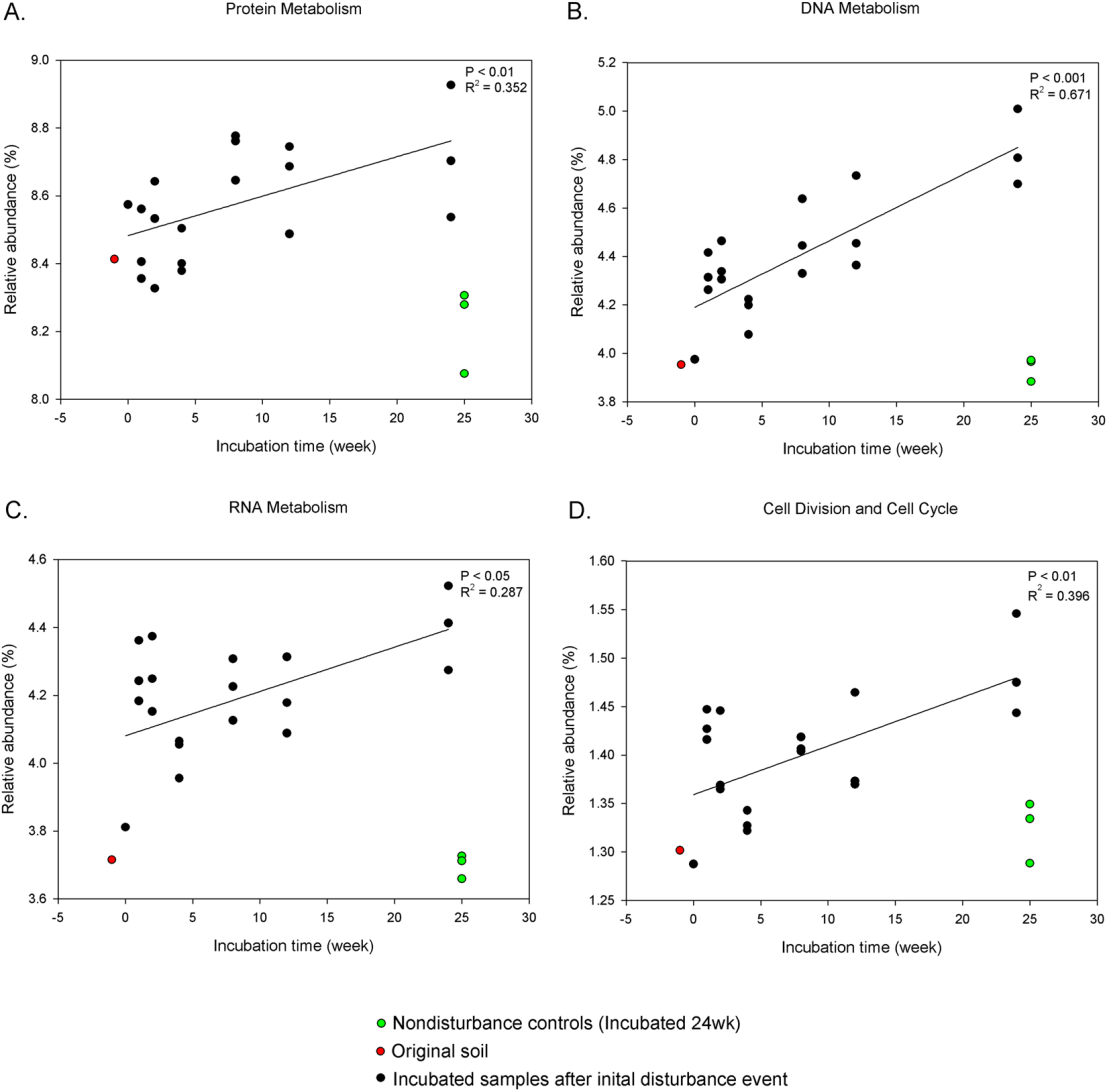
Relationship between incubation time and relative abundance of functional gene categories at subsystem level 1 (SEED database). Solid line represents linear regression fits to data. (**A**) Protein metabolism, (**B**) DNA metabolism, (**C**) RNA metabolism, (**D**) Cell division and cell cycle. Colored points were not included in the statistics.

**Figure 5:**
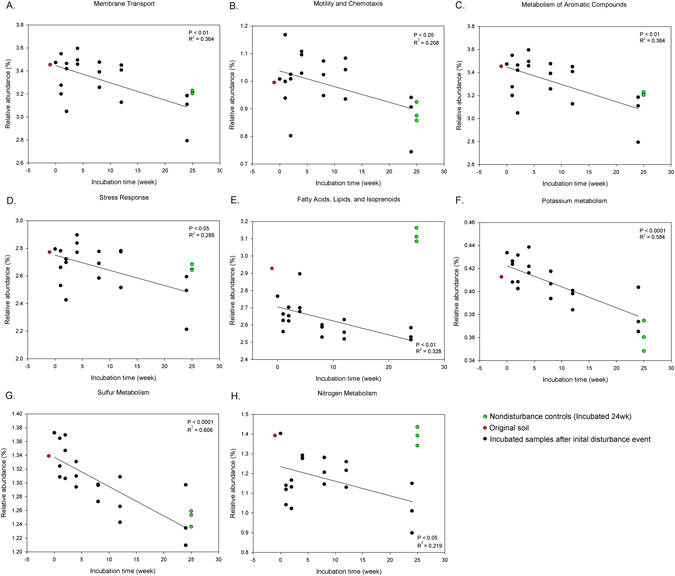
Relationship between incubation time and relative abundance of functional gene categories at subsystem level 1 (SEED database). (**A**) Membrane transport, (**B**) Motility and Chemotaxis, (**C**) Metabolism of Aromatic Compounds, (**D**) Stress Response, (**E**) Fatty Acids, Lipids and Isoprenoids, (**F**) Potassium metabolism, (**G**) Sulfur Metabolism, (**H**) Nitrogen Metabolism. Colored points were not included in the statistics.

Of the 28 functional gene categories, 14 categories differed significantly (P < 0.05) in relation to time since disturbance (Figs 4 and 5). At Functional Level 1, 6 gene categories which represent ‘housekeeping’^52^ genes related to protein metabolism (P < 0.01, R² = 0.352, Fig. 4A), DNA metabolism (P < 0.001, R² = 0.671, Fig. 4B), RNA metabolism (P < 0.05, R² = 0.287, Fig. 4C), and cell division and cell cycle (P < 0.01, R² = 0.396, Fig. 4D) all showed an increase in relative abundance over time (Fig. 4).

More narrowly defined functions classified as Level 2 under protein, RNA metabolism did not show any significant relationship with time since disturbance (P > 0.05), and showed this trend only when combined on Level 1 (Table 1). However, 5 separate functions classified under DNA metabolism showed increasing trends over time (Table 1).

**Table 1:** Functional abundance analysis of level 2 under functional gene level 1 (SEED database) which had increasing correlation with incubation time.

Function classification	Function classification	Regression		
Level 1	Level 2	P	R²	Coefficient
Protein Metabolism	—	—	—	—
DNA Metabolism	DNA repair	<0.001	0.571	0.00925
	DNA Metabolism	0.001	0.470	0.00596
	DNA replication	<0.001	0.687	0.00892
	DNA recombination	<0.001	0.519	0.00078
	CRISPs	<0.001	0.760	0.00126
RNA Metabolism	—	—	—	—
Clustering-based subsystems	recX and regulatory cluster	0.003	0.406	−0.00020
	Shiga toxin cluster	0.004	0.390	0.00062
	Two related proteases	0.040	0.226	0.00043
	Lysine Biosynthesis	0.042	0.221	−0.00229
	Tricarboxylate transporter	0.007	0.354	−0.00148
	Putrescine/GABA utilization cluster-temporal, to add to SSs	0.006	0.367	0.00270
	Ribosome-related cluster	0.001	0.464	0.00056
	D-tyrosyl-tRNA (Tyr) deacylase (EC 3.1.) cluster	0.007	0.357	0.00080
	Carbohydrates - #1	<0.001	0.753	0.00031
	DNA polymerase III epsilon cluster	0.011	0.322	−0.00033
	Pyruvate kinase associated cluster	0.004	0.396	0.00280
	Cytochrome biogenesis	0.046	0.215	0.00151
	Probably GTP or GMP signaling related	0.011	0.326	−0.00138
	Fatty acid metabolic cluster	0.040	0.225	0.00148
	Chemotaxis, response regulators	<0.001	0.661	0.00061
	Hypothetical protein possible functionally linked with Alanyl-tRNA synthetase	0.007	0.356	−0.00107
	Molybdopterin oxidoreductase	0.001	0.497	0.00028
	Sulfatases and sulfatase modifying factor 1 (and a hypothetical)	0.001	0.460	−0.00143
	Hypothetical associated with RecF	0.038	0.229	0.00026
	Probably Pyrimidine biosynthesis-related	0.001	0.494	0.00052
	TldD cluster	0.004	0.388	0.01092
	Clustering-based subsystems	0.006	0.365	0.00399
Cell Division and Cell Cycle	Cell Division and Cell Cycle	0.006	0.365	0.00399

Only classification levels having P < 0.05 were shown.

At Functional Level 1 using the SEED database, 8 gene categories related to membrane transport (P < 0.01, R² = 0.364, Fig. 5A), motility and chemotaxis (P < 0.05, R² = 0.208, Fig. 5B), metabolism of aromatic compounds (P < 0.001, R² = 0.364, Fig. 5C), stress response (P < 0.05, R² = 0.288, Fig. 5D), fatty acids, lipids and Isoprenoids (P < 0.01, R² = 0.328, Fig. 5E), potassium metabolism (P < 0.001, R² = 0.584, Fig. 5F), sulfur metabolism (P < 0.001, R² = 0.606, Fig. 5G), nitrogen Metabolism (P < 0.05, R² = 0.219, Fig. 5H) showed declining relative abundance over time (Fig. 5). Detailed functions classified as level 2 under nitrogen metabolism did not have a significant relationship with time (P > 0.05) and had trend only as combined on level 1 (Table 2). Under motility and chemotaxis, functional level 2 classification of social motility and nonflagellar swimming in bacteria and flagellar motility in prokaryota showed significantly (P < 0.05) decreasing trends after the initial disturbance event. Functional level 2 genes for heat shock responses, oxidative stress, acid stress, and stress response also showed decreasing patterns under the broader stress response label.

**Table 2:** Functional abundance analysis of level 2 under functional gene level 1 (SEED database) which had decreasing correlation with incubation time.

Function classification	Function classification	Regression		
Level 1	Level 2	P	R²	Coefficient
Membrane transport	Protein translocation across cytoplasmic membrane	0.007	0.352	0.00182
	Protein secretion system, Type V	<0.001	0.569	−0.00016
	Membrane Transport	0.011	0.321	−0.00703
	Protein secretion system, Type III	0.016	0.297	−0.00022
	Protein secretion system, Type VII (Chaperone/Usher pathway, CU)	0.005	0.384	−0.00011
Motility and Chemotaxis	Social motility and nonflagellar swimming in bacteria	0.008	0.351	−0.00004
	Flagellar motility in Prokaryota	0.024	0.264	−0.00469
Metabolism of Aromatic Compounds	Metabolism of central aromatic intermediates	<0.001	0.775	−0.00624
	Peripheral pathways for catabolism of aromatic compounds	0.002	0.437	−0.00768
	Metabolism of Aromatic Compounds	0.001	0.482	−0.00272
Stress Response	Heat shock	0.020	0.278	0.00166
	Oxidative stress - #1	0.006	0.364	−0.00620
	Acid stress	0.034	0.238	−0.00062
	Stress Response	<0.001	0.661	−0.00279
Fatty Acids, Lipids and Isoprenoids	Phospholipids	0.010	0.333	−0.00197
	Fatty Acids, Lipids, and Isoprenoids	0.014	0.307	−0.00303
	Fatty acids	0.015	0.299	−0.00554
	Triacylglycerols	0.010	0.331	−0.00012
	Isoprenoids	0.049	0.209	0.00249
Potassium metabolism	Potassium metabolism	<0.001	0.588	−0.00173
Sulfur Metabolism	Organic sulfur assimilation	0.003	0.413	−0.00313
Nitrogen Metabolism	—	—	—	—

Only classification levels having P < 0.05 were shown.

Since the abundance of Archaea increased after the initial disturbance event (Fig. 1B), we analysed corresponding Aachaeal functional genes through time (Supplementary Table S5) to understand what functional traits of Archaea were being selected for. At functional Level 1, 28 functional genes were classified. Among these 28 functions, 16 Archaeal functions showed significant changes over time since disturbance (P < 0.05) with a linear regression. In total, 5 of these Archaeal functional genes that showed trends over time were related to housekeeping functions. AmoA abundance did not show any trend over time.

## Discussion

There were clear time related trends of relative abundance in taxonomic profiles (Fig. 1) and functional genes (Figs 4 and 5). Also, composition of communities, based upon assignment of the metagenome reads and bacterial OTUs, showed that the microbial community progressively changed over time, initially in the general direction of the initial soil although it is unclear where the trajectory would ultimately arrive at if given long enough (Fig. 2).

As predicted, the early successional systems tended to have greater relative abundance of genes associated with motility (Fig. 5B). Extreme motility is a characteristic utilized by many early successional organisms, for example plants which rely on wind dispersal of seeds, or plants which rely on birds consuming berries and then dropping the seeds^53, 54^. In later succession of classical ecological systems involving plants, seaweeds and corals, the necessity of motility is reduced when *in situ* growth and persistence of mature individuals is more important than dispersal mechanisms of juveniles^55^. Active (as opposed to passive) motility in microorganisms takes various forms^56, 57^, but in the functional gene category characterized here it is dominated by genes for both flagellar and nonflagellar swimming, mainly in the bacteria which dominate this system (Table 2). Social motility is one of well-characterized group activities of bacterial systems^58, 59^. Surface-induced cooperative motilities are known to be widespread among bacteria^60–63^. The overall trend in relative abundance of this gene category – early abundance followed by later decline - may indicate that analogous to macroecological systems, this characteristic is also important on the much smaller spatial and temporal scale of succession experienced by microbial communities in soil. This finding provides interesting confirmation of the importance of similar traits during analogous stages in ecological succession across a vast range of spatial and temporal scales.

Greater relative abundance of genes related to basic ‘housekeeping’^52^ functions was predicted for the earlier stages of successional recovery of the soil system. However, we found the opposite pattern: these housekeeping genes, which relate to core metabolic functions were more abundant in late successional stages (Fig. 4A,B and C). Increase of those basic metabolisms may be related to increased importance of cell division and cell cycle (Fig. 4D). Possibly, after the early successional stage that favours dispersal through high motility functions, those and other gene categories become extraneous and there is more emphasis on core functions related to growth and cell division. However, the broad category of housekeeping genes includes functions on ‘amino acids and derivatives’, ‘cofactors, vitamins, prosthetic groups, pigments’, ‘nucleosides and nucleotides’, ‘protein metabolism’. Taken individually, these heterogenous functions showed varying trends.

We had predicted increased abundance of stress response genes in later succession, in relation to the lower nutrient availability, and increased interference competition. The main gene families identified under this category by MG-RAST were responses to oxidative stress, involved in producing antioxidants such as glutathione and in protection from reactive oxygen species. However, contrary to our prediction, there was a clear trend in the opposite direction: stress response genes were more abundant in the earliest time slices after disturbance and became progressively less common over time (Fig. 5D). It is unclear what it is about the earliest stages of succession in this system that leads to more of these stress response genes, or whether this really means that the early successional environment is in any meaningful way ‘stressful’. In successional systems involving larger organisms, connectivity and mutualism are thought to increase over time as the system ‘matures’^6^ – by extension, in our microcosm successional system it is possible that stressful aspects of the early succession might be lack of connectivity and support networks due to longer cell-cell spacing, or some particular aspect of colonizing soil or organic matter particles that are devoid of living cells. One possibility is osmotic stress caused by release of ions and other solutes from dead cells, killed during autoclaving.

Also contrary to predictions, there were no significant differences in relative abundance of cell signaling and virulence and defense genes over time. Genes relating to antibiotic resistance and to secondary compounds also showed no trend over time. In successional systems involving larger organisms, connectivity between organisms in both mutualistic and antagonistic interactions is seen as increasing in later succession^6^. However, if the groups of genes we pinpointed are any guide, it appears that such interactions are equally important throughout the successional time scale we studied.

We had predicted that viruses, and the corresponding CRISPR elements which defend against them, would become progressively more abundant over successional time. However, both showed no significant differences during the successional time series. It appears then that the community level importance and selective importance of viruses is not closely related to the processes of population and community succession, perhaps because of their high efficiency at spreading through the soil medium between susceptible hosts.

Plotted on an NMDS, there appears to be a clear and consistent progression in the composition of the total assemblage of functional genes (Fig. 2B), with the existence of a progression of distinct communities confirmed by ANOSIM. At first, in Week 1, the assemblage was the most different from the original (garden soil and time 0) soils, but over time it somewhat became more similar, at first returning in the general direction of the original soils but then moving away from them. By the end of the experiment, at week 24, rate of change appeared to become lower, although the time stage groups were still statistically distinct from one another according to the analysis of ANOSIM. The continuing difference from initial conditions at the end of the experiment stands in contrast with the control soil microcosms after 24 weeks which had not been through the 90% population reduction disturbance event, which still remained very close to the original soil on the NMDS.

The differences in functional gene and taxonomic community composition that are seen over time in our successional system, must be largely be brought about initially by the presence of a large volume of colonizable soil rich in nutrients from dead soil biota, and the opportunities and challenges presented by this. This system provides a potential analog for changes which might occur in nature or in agricultural systems following drastic disturbance events.

It appears that in our system (and possibly in systems most closely analogous to it nature), the timescale of soil ‘recovery’ to the original state, and ‘engineering resilience’ for biogeochemical functions will be of the order of years. This is similar to the findings of earlier studies of soil disturbance that measured biogeochemical processes rather than soil biota composition^64, 65^. Our study thus indicates how pervasive the effects of disturbance are in terms of functional genes, with a lack of complete recovery that likely persists on a timescale longer than we measured here. By contrast, less drastic physical disturbance of a soil (in our experiment, the initial sieving and then storage for a week at 3 °C) appears to have very little effect on soil functional gene profile after 24 weeks – as revealed by the soil’s resemblance to the initial state at time 0, and also its close resemblance to the original garden soil on day of harvest, even after a further 6 months’ incubation.

As in other studies of disturbance effects on both macroorganism and microorganism scales^3–5, 14, 22–24, 34^, we found a clear succession of communities of over time following disturbance (Fig. 2A,C). At the broadest level, bacteria became less abundant and Archaea became more abundant over time (Fig. 1A,B). The increase in Archaea over time may represent some unknown preadaptation to the late successional environment. When we analysed the trends in Archaeal genes by linear regression, there was no increase in the abundance of the Archaeal AmoA gene, contrasting with might have been expected given the prevailing view that most soil Archaea are ammonia oxidisers (Supplementary Table S5).

At the taxonomic level of phyla, amongst bacteria there was a shift in relative abundances from Proteobacteria towards Bacteroidetes over time after disturbance (Supplementary Table S3). This may partly relate to pH and TOC, since pH and TOC decreased slightly over the time sequence (Supplementary Table S2). With Envfit on the NMDS, in the context of explaining community composition, P of TOC was under 0.05 on the community in the taxonomic results from the shotgun metagenomic sequences based on M5NR taxonomic profile, while pH and TN influence the communities based on the bacterial OTU level (Supplementary Fig. S1A, Supplementary Fig. S1B). However, it is not clear that TOC was in itself directly responsible for the compositional changes, rather than merely being an incidental correlate.

There is a clear succession of the community composition of total soil biota (from all biotic kingdoms), as judged from the metagenome reads that can be assigned taxonomically (Fig. 2A). The NMDS plot shows that over time that, just as for bacteria, the ‘total biota’ of these disturbed communities at first shifts back in the general direction of the original soils. However, as time passed, the rate of change on the NMDS appeared to decrease, and by the end of the experiment, at week 24, the communities appeared to have followed a different trajectory and had not reached any point resembling the original state or that of the controls. A similar trend was also observed in composition of functional genes (Fig. 2B). The functional and taxonomic changes of the soil biota seen in this experiment provides a potential analog for changes which might occur in nature or agricultural systems following drastic disturbance events. Field-based studies also suggest a very long time scale for recovery of soil communities to their original state following fire disturbance – as much as a decade^67^.

Diversity at the identifiable species level from all kingdoms (based on the M5NR database) recovered after the initial disturbance event (Fig. 3A), unlike the functional diversity at different levels (Fig. 3B,C and D). At the 1-week stage after initial disturbance, total biota Shannon diversity was lower than on day 0, but increased until the 4-week stage (Fig. 3A). This species level diversity was then maintained unchanged until the end of experiment. Although taxonomic diversity recovered and maintained itself after the 4-week stage, throughout the experiment diversity of functional genes remained lower than day 0, and actually decreased over time (Fig. 3B,C and D).

Overall, our microcosm experiments (which exclude environmental changes) imply that microbial communities impacted by a drastic disturbance event change their functional profile (Figs 4 and 5) gradually over time, and are still different from undisturbed soil after a 6 month period.

As with all experimental systems (which are simplifications of nature), a question that must be addressed is whether this particular system is too far removed from reality to be relevant to understanding natural systems. We relied here on the principle that the main effect of autoclaving on nutrient release would be due to death and lysis of living cells. However, it is also possible that autoclaving significantly alters the state of soil humus, causing it to break down and release extra carbon and nutrients. Various experiments in the past have studied the effect of soil heating (including autoclaving) on nutrient release. For example, Serrasolas and Khanna^67^ studied the effect of soil heating and autoclaving on extractable nitrogen, N mineralisation and C metabolism by heating five forest soils in the laboratory at 60 °C, 120 °C and 250 °C. In other experiments on heated soils, an increase in easily extractable C, N and other elements has been observed, and increases in rapidly respirable C^38, 68–71^. As previous authors acknowledge, this release of C and N is probably due to a combination of: (a) Lysis of microbial cells. (b) Increase in the solubility of both humic and inorganic forms of N, (c) Partial ashing of organically bound nutrients at high temperatures. While the ‘ashing’ process is generally only seen as relevant to the high temperatures of 250 °C or above, a) and b) are indeed both possibilities at the autoclaving temperature of 120 °C. Nevertheless, there has apparently been no explicit study of the extent to which soil C and N release from soil humus, for example, contributes to the observed release of C and N. The distinct possibility remains that a large part of the ‘alteration’ of soil nutrient availability following previous studies of experimental soil heating is actually from death of soil biota, just as we had proposed here. In any case, if alteration and breakdown soil of humus does occur on autoclaving, it does not invalidate this experimental system as a representation of nature, as this would also occur during comparable heating of soils from wildfire or volcanic activity.

## Conclusions

Our study gives some perspectives on a commonplace, important, but little studied system – disturbed soil. The results revealed strong, predictable changes in both the taxonomic composition and functional gene profile of the soil biota, in response to a nonselective disturbance event that had killed most of the community.

Interestingly, it is apparent from this experiment that taxonomic diversity (as identified from the metagenome and the 16S rRNA amplicon-based community) in this system does not show a decreasing pattern with time as functional diversity does (as defined in terms of quantitative diversity of functions of genes present) (Fig. 3). This is in contrast with the indications of mesocosm/microcosm studies on larger organisms which have suggested that greater species diversity leads to greater empirical functional diversity – also leading to greater resilience^12^. Thus, for soil microbiota these two characteristics of the community which would be expected to be closely linked, are in fact decoupled.

In some respects, our miniature successional system agreed with our predictions based upon observation of ‘classic’ successions involving large organisms. For example, all the biotic characteristics of the soil changed in the general direction of a return towards the initial conditions before disturbance/control conditions of an incubated undisturbed soil, although recovery rate apparently reduced after 4 weeks. There is also a clear functional and taxonomic difference in the spectrum of occupied niches between ‘early’ and ‘later’ communities post-disturbance.

However, in most other respects our predictions based on analogy with successional systems of large organisms were not supported. In a sense, this is no great surprise, since the scales and biological affinities of the organisms involved are very different – and finding such differences may ultimately help to inform us of the true nature of soil ecological processes.

It is also clear that disturbance of the type we simulated here, involving death of the majority of the soil biota, has a drastic effect that persists months, and perhaps years - as the system had still not reached the original or control state by the end of this 6 month experiment.

It would also be very interesting to use similar microcosm systems to that we used here to study changes biogeochemical fluxes such as CO² and N mineralization, in relation to disturbance. This could enable linkage of the functional and diversity changes seen in an experimental system such as this to biogeochemical functions of the soil such as respiration, nitrogen mineralization, or the ability to metabolise pollutants. Our system might also offer a useful analog for restoration ecology, where by a developed soil rich in biota is mixed into a relatively sterile and undeveloped regolith such as mine tailings.

## Methods

### Preparation of soil microcosms for disturbance gradient study

The soil for this experiment was taken from an area approximately 30 m × 20 m in a fallow field (Supplementary Fig. S2, Supplementary Fig. S3) located in Suwon, South Korea (lat. 37°16′N, long. 126°59′E) on the University Farm of Seoul National University in mid-June 2015. Soil was taken from the top 10 cm with large roots and stones removed, and it was thoroughly homogenized by sieving through a 2-mm mesh. The experiment itself was conducted in the laboratories of the School of Biological Sciences of Seoul National University. The sieved soil was sterilized by autoclaving twice for 1 h each time at 121 °C, to provide a large pool of sterile soil for ‘restocking’ pots following disturbance. Soil sterility was validated by enumeration of heterotrophic bacteria by the most probable number (MPN) technique^72, 73^, and absence of significant quantities of intact DNA by failure of test PCR for bacteria 16S and fungal ITS. Soil was frozen at −20 °C until ready for the experiment to begin.

Each pot in the experimental system was initially stocked with 450 g of moist sterilized soil and 50 g of moist unsterilized sieved soil from the same batch, which has been stored at 3 °C for one week prior to the experiment. This was intended to simulate a ‘disturbance’ event in which 90% of soil biota had been killed, with the remaining 10% going on to recolonize the whole bulk of the soil. The sterilized and unsterilized soil were thoroughly mixed by shaking in a sterile bag before being placed in the pot.

We set three replicates of disturbed and incubated controls with different incubation times: 1-week, 2-week, 4-week, 8-week, 12-week and 24-week and non-disturbance control incubated 24-week. One 0-week sample was collected after the disturbance event. In total 22 pots (15 × 15 × 13 cm, ca. 500 g of soil each) were arranged in a completely randomized design with each replicates. Each soil-filled pot was placed with another empty pot upside down over it, forming a lid that was sealed around the edges with tape, to avoid as much as possible additional recruitment from direct cross contamination by dust from adjacent pots. Five holes (each 0.5 mm in diameter) were drilled in the top of each pot, enough to allow aeration but unlikely to allow soil dust to travel between pots.

The pots were kept in a growth chamber in darkness at 24 °C and all were watered every week with a uniform 50 ml of sterile distilled water to maintain a moderately damp soil. At each watering time, all pots were switched around randomly in terms of their positions within the growth chamber. The experiment was continued for 24 weeks and soil samples for DNA extraction were be taken from the mixture of each pot.

### Chemical analysis and DNA extraction

Soil analyses were carried out at the National Instrumentation Center for Environmental Management (NICEM, South Korea). Soil pH, total nitrogen (TN) and total organic carbon (TOC) content were measured using standard protocols of the Soil Science Association of America (SSSA).

Total DNA was extracted from all the samples (from both disturbance and culture study) using the Power Soil DNA extraction kit (MoBio Laboratories, Carlsbad, CA, USA) following manufacturer’s instructions and stored at −20 °C until further processing.

### Metagenomic sequencing and bioinformatics analyses

All the purified DNA samples were sequenced using a Nextseq500 paired-end of 2 × 150 bp (Illumina) at Celemics Incorporation (Seoul, Korea). In total 71,263,671 reads of sequence data were generated. Paired end metagenome sequences were annotated with the Metagenomics Rapid Annotation (MG-RAST) pipeline version 3.3^74^. Phylogenetic information was extracted from the metagenomes using M5NR data bases using BLASTX (e-value less than 1 × 10^−5^, minimum percent identity was 60% and sequence match length greater than 15 nucleotides). Functional profiles were generated with the SEED subsystems database^75^ using a maximum e-value of 1e-5, a minimum identity of 60%, and a minimum alignment length of 15 aa. These profiles were then normalized for differences in sequencing coverage by calculating percent distribution, prior to downstream statistical analysis. All of the sequence data are available under the MG-RAST project ID 18857and 18393 (http://metagenomics.anl.gov/linkin.cgi?project=18857, http://metagenomics.anl.gov/linkin.cgi?project=18393).

To do a 16S rRNA amplicon sequencing, total DNA was extracted from all of 23 samples using the Power Soil DNA extraction kit (Mo Bio Laboratories, Carlsbad, CA, USA) following manufacturer’s instructions. DNA was PCR amplified for the V3 region of bacterial 16S rRNA and paired end sequenced (2 × 300 bp) by a Illumina MiSeq from Centre for Comparative Genomics and Evolutionary Bioinformatics (CGEB), Dalhousie University (Halifax, Canada). Totally sequence count 693,659 of sequence data were generated. Paired sequences were assembled and further sequence processing was performed following the Miseq SOP (http://www.mothur.org/wiki/MiSeq_SOP) and singletons were removed using the Mothur^74^. Taxonomy was assigned using EzTaxon database^76^ using the classify command in Mothur. For operational taxonomic unit (OTU)-based analysis, distances between sequences were calculated. Sequences which have over 97% similarity were merged into an OTU and 11,720 OTUs were found. Each sample was subsampled to 6,781 reads to calculate unweighted UniFrac matrix. 16S rRNA amplicon sequence data are available under the MG-RAST project ID 21542 (http://metagenomics.anl.gov/linkin.cgi?project=mgp21542).

A nonmetric multidimensional scaling (NMDS) plot was used to visualize the structure among samples, using the taxonomic and functional abundance matrix using PRIMER v6. The plots were generated from Bray–Curtis similarity index matrices (M5NR taxonomic profile at family level, SEED subsystem function at level 3 and bacterial 16S rRNA gene at OTU level). ANOSIM analysis was done by PRIMER v6 and the maximum number of permutations was 999.

Diversity measurements (Shannon’s index) were calculated based on M5NR taxonomy at species level as concept of diversity is based on species level and SEED subsystem function at level 3 by using software R version 3.1.2^77^. We plotted Envfit with the content of soil parameters and diversity of each samples and mapped sampling site (Supplementary Fig. S2) by using software R version 3.1.2. Software SigmaPlot 10.0 was used for regression study of Shannon diversity index on each treatments.

### Data Accessibility

All of the sequence data are available under the MG-RAST project ID 18857, 18393 and 21542 (http://metagenomics.anl.gov/linkin.cgi?project=18857, http://metagenomics.anl.gov/linkin.cgi?project=18393, http://metagenomics.anl.gov/linkin.cgi?project=mgp21542).

## Electronic supplementary material

Supplementary Information
