# Supplementary material for: Effect of experimental soil disturbance and recovery on structure and function of soil community: a metagenomic and metagenetic approach
Source: Sci Rep. 2017 May 23;7:2260. doi: 10.1038/s41598-017-02262-6 (PMC5442152; doi:10.1038/s41598-017-02262-6)
Supplement: Supplementary file 1 — Supplementary Information [file 41598_2017_2262_MOESM1_ESM.pdf]

Supplementary Information

Effect of experimental soil disturbance and recovery on structure and function of soil community: a metagenomic and metagenetic approach.

Soobeom Choi[1], Hokyung Song[1], Binu M. Tripathi[2], Dorsaf kerfahi[1], Hyoki Kim[3], Jonathan M. Adams[1]*

[1]Department of Biological Sciences, College of Natural Sciences, Seoul National University, Gwanak-Gu, Seoul, 08826, Republic of Korea

[2]Arctic Research Center, Korea Polar Research Institute, Icheon-si, Gyeonggi-do, 21990, Republic of Korea

[3]Celemics Inc., 371-17, Gasan-dong, Geumcheongu, Seoul, 153-718, Republic of Korea

*** Corresponding author** foundinkualalumpur@yahoo.com

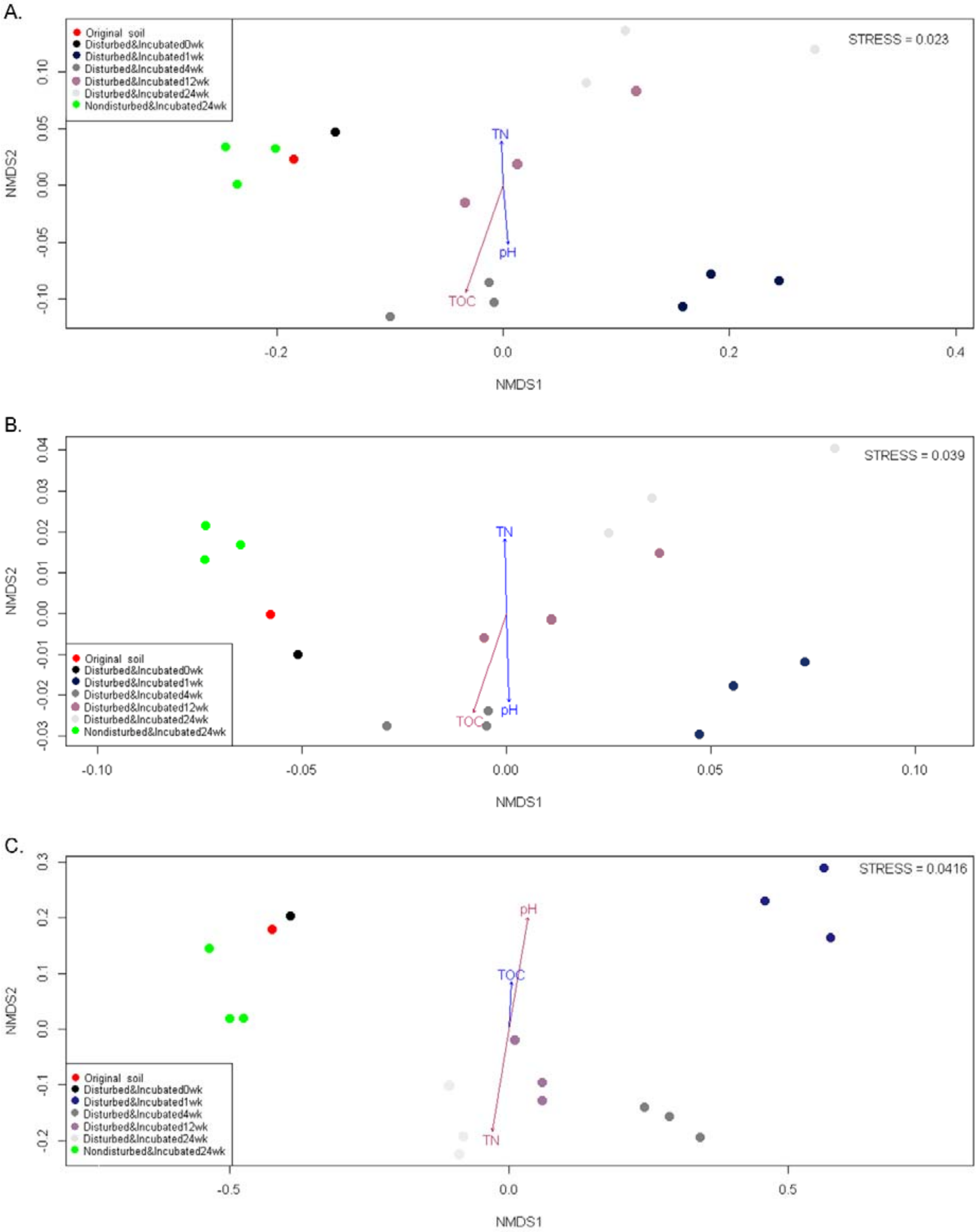

Supplementary fig. S1. NMDS plot generated using weighted pairwise Unifrac distances between samples and Envfit with pH, TOC and TN. Environmental factor which had P < 0.05 marked red.

A. NMDS plot and Envfit of shotgun metagenomic sequences based on M5NR taxonomic profile at family level, B. NMDS plot and Envfit of shotgun metagenomic sequences based on SEED functional profile at subsystem level 3, C. NMDS plot and Envfit of OTU level.

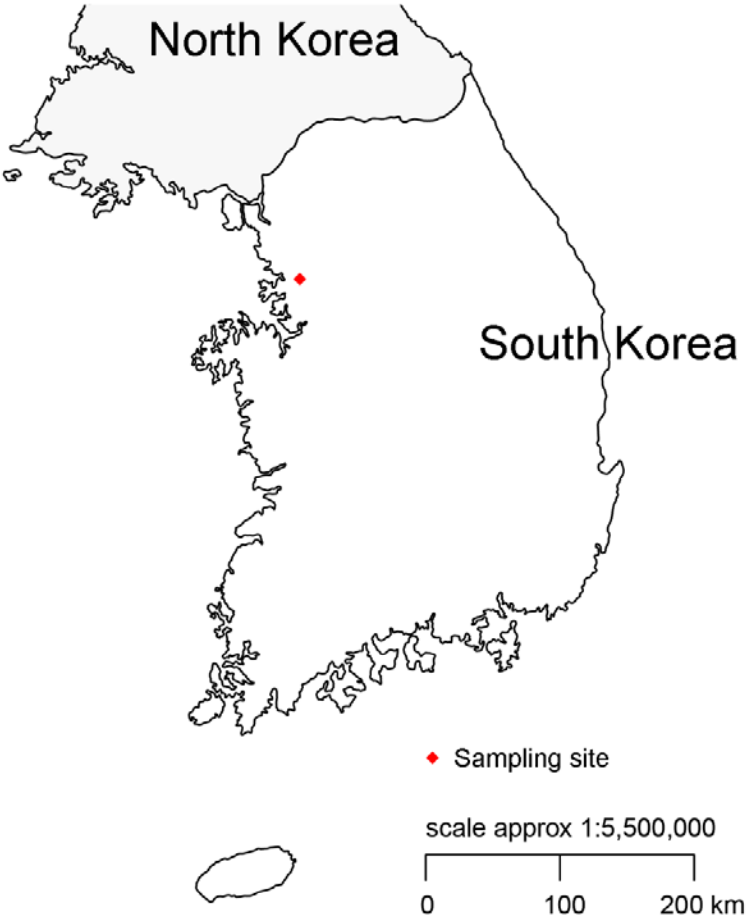

Supplementary fig. S2. A map of sampling area, University Farm of Seoul National University, Suwon, South Korea. Samples marked as red. This map was generated by using software R version 3.1.2.

Latitude: 37°16'N, Longitude: 126°59'E

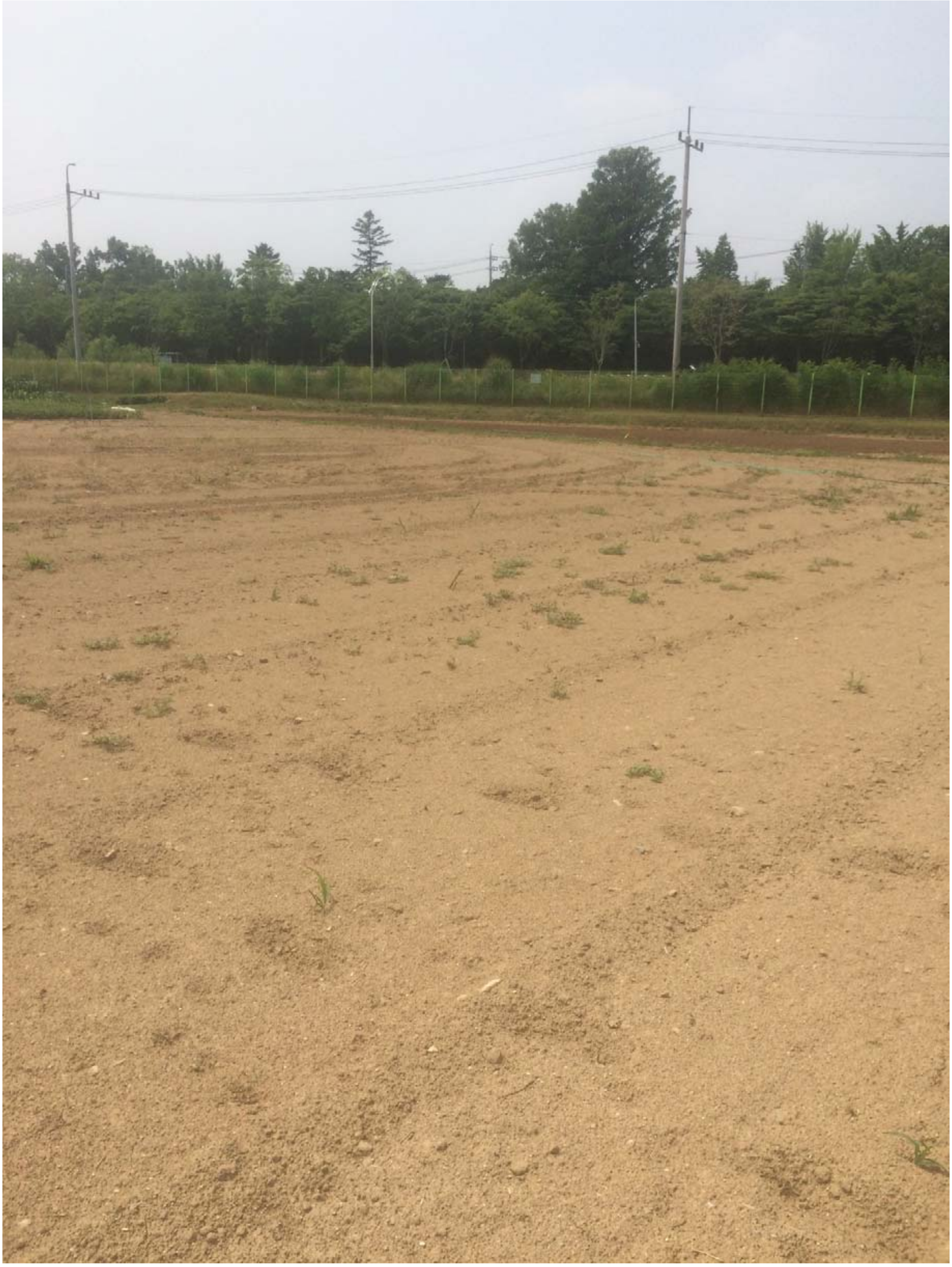

Supplementary fig. S3. Photo of the sampling site.

Supplementary table S1. Total number of base pairs, sequencing reads.

MG-RAST ID	Sample name	Treatment	Sampling date	Total bp	Sequence count	Predicted proteins	Annotated proteins (%)
4707578.3	OR	Original soil	2015-06-16	627969701	3473797	2367047	37.41
4707563.3	DC0	Disturbed&Incubated0wk	2015-07-07	572161561	3204375	2198562	27.7
4707570.3	DC1_1	Disturbed&Incubated1wk	2015-07-14	575197473	3114726	2113170	39.44
4707583.3	DC1_2	Disturbed&Incubated1wk	2015-07-14	554356262	3093279	2048950	39.58
4707561.3	DC1_3	Disturbed&Incubated1wk	2015-07-14	545495218	3018065	1976633	38.14
4707566.3	DC2_1	Disturbed&Incubated2wk	2015-07-21	601812503	3274033	2145887	31.7
4707564.3	DC2_2	Disturbed&Incubated2wk	2015-07-21	570773518	3155286	2120126	40.64
4707582.3	DC2_3	Disturbed&Incubated2wk	2015-07-21	552567024	3026986	2032050	31.4
4707581.3	DC4_1	Disturbed&Incubated4wk	2015-08-04	652609696	3637570	2558238	41.92
4707574.3	DC4_2	Disturbed&Incubated4wk	2015-08-04	538489571	2992906	2078311	32.9
4707589.3	DC4_3	Disturbed&Incubated4wk	2015-08-04	691109486	3855896	2652703	41.28
4707584.3	DC8_1	Disturbed&Incubated8wk	2015-09-01	582908908	3257066	2226347	28.9
4707577.3	DC8_2	Disturbed&Incubated8wk	2015-09-01	609806249	3412911	2366742	28.1
4707572.3	DC8_3	Disturbed&Incubated8wk	2015-09-01	652423120	3660170	2532948	29.5
4707568.3	DC12_1	Disturbed&Incubated12wk	2015-09-29	676225075	3762861	2570042	37.52
4707585.3	DC12_2	Disturbed&Incubated12wk	2015-09-29	643579358	3497651	2447951	37.7
4707580.3	DC12_3	Disturbed&Incubated12wk	2015-09-29	650301895	3553533	2457049	33.58
4707567.3	DC24_1	Disturbed&Incubated24wk	2015-12-22	566112460	3108174	2027623	34.65
4707565.3	DC24_2	Disturbed&Incubated24wk	2015-12-22	634245205	3399243	2034141	37.23
4707576.3	DC24_3	Disturbed&Incubated24wk	2015-12-22	580058103	3246637	2218102	30.85
4702982.3	NC1	Nondisturbed&Incubated24wk	2015-12-22	251762454	1416604	955073	34.82
4702995.3	NC2	Nondisturbed&Incubated24wk	2015-12-22	295991344	1644333	1145926	35.92
4702971.3	NC3	Nondisturbed&Incubated24wk	2015-12-22	263241389	1457569	1008593	33.18

Supplementary table S2. Results of soil chemical analysis.

Sample name	Treatment	pH	TOC (%)	TN (%)
OR	Original soil	6.7	1.59	0.157
NC1	Nondisturbed&Incubated24wk	6.0	1.45	0.179
NC2	Nondisturbed&Incubated24wk	5.9	1.54	0.185
NC3	Nondisturbed&Incubated24wk	6.6	1.49	0.162
DC0	Disturbed&Incubated0wk	6.7	1.46	0.155
DC1_1	Disturbed&Incubated1wk	6.6	1.46	0.159
DC1_2	Disturbed&Incubated1wk	6.7	1.51	0.160
DC1_3	Disturbed&Incubated1wk	6.6	1.52	0.156
DC4_1	Disturbed&Incubated4wk	6.4	1.59	0.163
DC4_2	Disturbed&Incubated4wk	6.3	1.48	0.172
DC4_3	Disturbed&Incubated4wk	6.3	1.48	0.177
DC12_1	Disturbed&Incubated12wk	6.3	1.54	0.167
DC12_2	Disturbed&Incubated12wk	6.3	1.48	0.163
DC12_3	Disturbed&Incubated12wk	6.3	1.43	0.167
DC24_1	Disturbed&Incubated24wk	5.9	1.38	0.190
DC24_2	Disturbed&Incubated24wk	5.9	1.42	0.189
DC24_3	Disturbed&Incubated24wk	6.5	1.43	0.156
**Regression**		P=0.0099, R^2=0.502, Coefficient:-0.01907	P=0.0155, R^2=0.459, Coefficient:-0.00423	-

Supplementary table S3. Relative abundance (%) (means ± SD) of bacterial phyla observed in shotgun metagenomics sequences.

phylum	Original soil	Nondisturbed control	Incubation control time (weeks)							Regression	Coefficient
			0	1	2	4	8	12	24		
Poribacteria	0.07	0.08 ± 0.03	0.08	0.04 ± 0.01	0.02 ± 0	0.07 ± 0.01	0.14 ± 0.01	0.17 ± 0.06	0.36 ± 0.18	$P<0.0001$, $R^2=0.740$	0.0141
Dictyoglomi	0.06	0.05 ± 0.02	0.07	0.04 ± 0	0.04 ± 0	0.04 ± 0	0.05 ± 0.01	0.06 ± 0.01	0.07 ± 0.01	$P<0.001$, $R^2=0.586$	0.0015
Deinococcus-Thermus	0.82	0.71 ± 0.29	0.81	0.34 ± 0.02	0.35 ± 0.03	0.48 ± 0.05	0.54 ± 0.02	0.56 ± 0.05	0.53 ± 0.15	x	
Gemmatimonadetes	1.24	0.95 ± 0.46	1.17	0.21 ± 0.05	0.24 ± 0.09	1.36 ± 0.21	0.96 ± 0.31	1.19 ± 0.57	0.57 ± 0.39	x	
Firmicutes	6.08	5.09 ± 2.27	6.56	19.2 ± 4.32	15.63 ± 6.39	5.77 ± 0.6	9.54 ± 1.9	13.05 ± 4.44	16.74 ± 5.44	x	
Fusobacteria	0.09	0.07 ± 0.03	0.1	0.11 ± 0.01	0.09 ± 0.01	0.07 ± 0.01	0.11 ± 0.01	0.11 ± 0.03	0.15 ± 0.02	$P<0.001$, $R^2=0.552$	0.0025
Cyanobacteria	2.64	1.90 ± 0.83	2.8	1.5 ± 0.06	1.42 ± 0.05	1.58 ± 0.07	2.08 ± 0.14	2.14 ± 0.16	2.31 ± 0.4	$P=0.012$, $R^2=0.316$	0.0304
Deferribacteres	0.09	0.07 ± 0.03	0.1	0.05 ± 0	0.05 ± 0	0.06 ± 0.01	0.09 ± 0.01	0.09 ± 0.01	0.11 ± 0.01	$P<0.0001$, $R^2=0.619$	0.0024
Chrysiogenetes	0.03	0.02 ± 0.01	0.03	0.02 ± 0	0.01 ± 0	0.02 ± 0	0.03 ± 0	0.03 ± 0	0.03 ± 0	$P<0.01$, $R^2=0.391$	0.0005
Acidobacteria	5.47	3.48 ± 1.37	5.65	2.09 ± 0.23	1.99 ± 0.41	3.2 ± 0.22	4.21 ± 0.24	4.08 ± 0.39	2.92 ± 0.98	x	
Spirochaetes	0.27	0.21 ± 0.10	0.3	0.26 ± 0.02	0.26 ± 0.01	0.23 ± 0.02	0.33 ± 0.03	0.31 ± 0.04	0.39 ± 0.04	$P<0.0001$, $R^2=0.668$	0.0060
Nitrospirae	0.66	0.69 ± 0.22	0.73	0.3 ± 0.04	0.22 ± 0.04	0.66 ± 0.12	1.19 ± 0.1	1.41 ± 0.48	2.64 ± 1.19	$P<0.0001$, $R^2=0.758$	0.1001
Chlamydiae	0.12	0.10 ± 0.05	0.14	0.09 ± 0.01	0.08 ± 0	0.09 ± 0.01	0.18 ± 0.01	0.21 ± 0.07	0.26 ± 0.04	$P<0.0001$, $R^2=0.733$	0.0080
Tenericutes	0.03	0.03 ± 0.01	0.04	0.03 ± 0	0.03 ± 0	0.02 ± 0	0.04 ± 0	0.04 ± 0.01	0.05 ± 0.01	$P<0.001$, $R^2=0.536$	0.0011
Fibrobacteres	0.02	0.01 ± 0.01	0.02	0.02 ± 0	0.02 ± 0	0.02 ± 0	0.02 ± 0	0.02 ± 0	0.02 ± 0	$P<0.001$, $R^2=0.331$	0.0003
Verrucomicrobia	2.97	1.69 ± 0.83	3.38	1.71 ± 0.21	1.71 ± 0.6	2.77 ± 0.44	3.47 ± 0.42	3.08 ± 0.28	2.75 ± 1.18	x	
Chlorobi	0.53	0.38 ± 0.17	0.58	0.53 ± 0.03	0.54 ± 0.03	0.49 ± 0.04	0.67 ± 0.06	0.66 ± 0.08	0.77 ± 0.03	$P<0.0001$, R	0.0109

										^{2}=0.695	
Lentisphaerae	0.07	0.05 ± 0.02	0.08	0.05 ± 0	0.05 ± 0.01	0.07 ± 0	0.09 ± 0.01	0.08 ± 0	0.08 ± 0.02	P=0.018, R^2=0.286	0.0012
Aquificae	0.22	0.18 ± 0.07	0.22	0.14 ± 0	0.13 ± 0	0.16 ± 0.01	0.23 ± 0.02	0.24 ± 0.02	0.3 ± 0.02	P<0.0001, R^2=0.784	0.0068
Proteobacteria	45.18	36.21±13.78	44.45	31.99 ± 5.87	31.43 ± 9.25	52.36 ± 1.62	39.06 ± 7.14	38.3 ± 7.56	28.12 ± 8.07	x	
Elusimicrobia	0.03	0.02 ± 0.01	0.03	0.02 ± 0	0.02 ± 0	0.02 ± 0	0.03 ± 0	0.03 ± 0	0.04 ± 0.01	P<0.001, R^2=0.503	0.0007
Actinobacteria	18.08	19.33±8.49	15.12	3.96 ± 1.01	5.77 ± 1.75	8.19 ± 3.25	6.03 ± 1.51	6.06 ± 2.4	3.6 ± 1.21	x	
Chloroflexi	4.56	3.56 ± 1.62	4.52	1.41 ± 0.11	1.33 ± 0.15	1.65 ± 0.12	2.15 ± 0.15	2.38 ± 0.1	2.65 ± 1.03	x	
Planctomycetes	3.91	2.43 ± 1.20	4.17	1.25 ± 0.18	1.27 ± 0.41	3.26 ± 0.08	3.93 ± 0.21	3.55 ± 0.72	2.57 ± 1.26	x	
Synergistetes	0.13	0.11 ± 0.05	0.12	0.06 ± 0	0.05 ± 0	0.07 ± 0.01	0.09 ± 0.01	0.1 ± 0	0.1 ± 0.03	P=0.018, R^2=0.289	0.0016
Bacteroidetes	5.53	3.09 ± 1.45	7.56	33.83 ± 4.53	36.47 ± 6.34	16.57 ± 3.99	23.81 ± 7.53	21.09 ± 6.75	30.72 ± 7.9	x	
Thermotogae	0.22	0.18 ± 0.08	0.13	0.12 ± 0	0.12 ± 0	0.13 ± 0.02	0.17 ± 0.01	0.22 ± 0.04	0.19 ± 0.01	P<0.001, R^2=0.519	0.0037

Supplementary table S4. Relative abundance (%) (means ± SD) of bacterial phyla observed in bacterial 16S rRNA amplicon sequences.

phylum	Original soil	Nondisturbed control	Incubation control time						
			0	1	2	4	8	12	24
AD3	0	0 ± 0	0.01	0 ± 0	0 ± 0	0 ± 0	0 ± 0	0 ± 0	0.01 ± 0.01
AF234118_p	0	0 ± 0	0.03	0 ± 0	0 ± 0	0 ± 0	0 ± 0.01	0 ± 0	0 ± 0.01
AY435510_p	0	0 ± 0	0	0 ± 0	0 ± 0	0 ± 0	0 ± 0	0 ± 0	0 ± 0
Acidobacteria	9.75	8.53 ± 2.23	9.33	1.03 ± 0.14	0.83 ± 0.21	2.57 ± 0.33	4.23 ± 0.32	4.28 ± 0.41	4.65 ± 1.44
Actinobacteria	23.85	24.49 ± 3.76	26.13	12.88 ± 1.11	21.87 ± 3.22	9.29 ± 1.01	14.61 ± 1.25	15.94 ± 1.11	18.46 ± 0.24
Armatimonadetes	0.07	0.08 ± 0.04	0.04	0.01 ± 0.01	0.03 ± 0.03	0.19 ± 0.05	0.17 ± 0.09	0.15 ± 0.05	0.16 ± 0.01
BRC1	0	0.03 ± 0.03	0.06	0.01 ± 0.01	0.01 ± 0.01	0.03 ± 0.02	0.12 ± 0.04	0.06 ± 0.03	0.07 ± 0.02
Bacteroidetes	8.01	4.96 ± 1.48	7.83	13.95 ± 1.85	16.21 ± 0.55	22.60 ± 0.42	20.43 ± 2.87	16.38 ± 2.51	13.40 ± 1.81
Chlamydiae	0.22	0.10 ± 0.08	0.16	0.01 ± 0.01	0 ± 0.01	0.02 ± 0.01	0.21 ± 0.13	0.18 ± 0.07	0.12 ± 0.05
Chlorobi	0.01	0.07 ± 0.03	0.06	0 ± 0	0.01 ± 0.01	0.06 ± 0.04	0.06 ± 0.01	0.09 ± 0.05	0.11 ± 0.05
Chloroflexi	11.72	14.02 ± 2.66	10.53	1.17 ± 0.10	1.05 ± 0.04	2.21 ± 0.34	2.79 ± 0.22	3.51 ± 0.07	7.29 ± 0.79
Cyanobacteria	0.37	0.39 ± 0.08	0.19	0.12 ± 0.02	0.07 ± 0.02	0.40 ± 0.08	0.43 ± 0.14	0.44 ± 0.07	0.42 ± 0.03
DQ833500_p	0	0 ± 0	0.01	0 ± 0	0 ± 0	0 ± 0	0 ± 0	0 ± 0	0 ± 0
Deinococcus-Thermus	0	0 ± 0	0.03	0 ± 0	0 ± 0	0 ± 0	0 ± 0	0 ± 0	0 ± 0.01
Elusimicrobia	0.18	0.07 ± 0.03	0.16	0.01 ± 0.01	0.01 ± 0.01	0.02 ± 0.01	0.07 ± 0.03	0.11 ± 0.04	0.08 ± 0.03
Fibrobacteres	0.04	0.01 ± 0.02	0.03	0 ± 0.01	0 ± 0.01	0.02 ± 0.03	0.05 ± 0.03	0.06 ± 0.03	0.01 ± 0.01
Firmicutes	3.2	5.29 ± 0.87	3.5	6.83 ± 1.49	5.07 ± 0.99	4.81 ± 0.11	7.32 ± 1.58	11.42 ± 1.81	7.04 ± 1.43
GN02	0	0.01 ± 0.01	0	0 ± 0	0 ± 0	0 ± 0	0.02 ± 0.02	0 ± 0.01	0 ± 0
GN04	0	0 ± 0	0	0 ± 0.01	0 ± 0	0 ± 0	0 ± 0	0 ± 0	0 ± 0
Gemmatimonadetes	4.37	4.23 ± 1.38	4.51	0.64 ± 0.06	0.71 ± 0.21	5.15 ± 0.30	4.23 ± 0.41	5.96 ± 0.35	5.64 ± 1.17
MATCR	0.04	0.04 ± 0.16	0.03	0.00 ± 0.00	0.00 ± 0.00	0.00 ± 0.01	0.01 ± 0.01	0.02 ± 0.01	0.01 ± 0.01
NKB19	0.01	0 ± 0	0	0 ± 0	0 ± 0	0 ± 0.01	0 ± 0.01	0 ± 0	0 ± 0
Nitrospirae	0.74	2.81 ± 0.81	0.71	0.07 ± 0.01	0.04 ± 0.03	0.46 ± 0.09	0.76 ± 0.32	0.90 ± 0.17	2.41 ± 1.41
OD1	0	0.02 ± 0.02	0.01	0 ± 0	0 ± 0	0 ± 0.01	0.01 ± 0	0.02 ± 0.01	0.05 ± 0.03
OP11	0	0 ± 0.01	0.03	0 ± 0	0 ± 0	0 ± 0	0 ± 0	0 ± 0	0 ± 0
OP3	0	1 ± 0.01	0.01	0 ± 0.01	0 ± 0	0 ± 0	0.02 ± 0.02	0.01 ± 0.01	0.02 ± 0.03

Planctomycetes	1.45	0.97 ± 0.13	1.19	0.31 ± 0.05	0.27 ± 0.11	1.71 ± 0.28	1.58 ± 0.50	1.28 ± 0.09	1.20 ± 0.33
Proteobacteria	31.53	30.45 ± 1.74	30.94	61.86 ± 3.44	52.67 ± 3.25	47.80 ± 1.86	39.30 ± 0.57	35.43 ± 0.17	35.29 ± 1.99
TM6	0	0.02 ± 0.02	0.04	0 ± 0.01	0 ± 0	0 ± 0.01	0.03 ± 0.04	0.02 ± 0.01	0.01 ± 0.02
TM7	0.01	0.05 ± 0.02	0.06	0 ± 0.01	0 ± 0.01	0 ± 0.01	0.03 ± 0.01	0.04 ± 0.02	0.07 ± 0.02
Tenericutes	0	0 ± 0.01	0	0 ± 0	0 ± 0	0 ± 0	0 ± 0.01	0 ± 0	0 ± 0.01
Verrucomicrobia	3.05	2.09 ± 0.63	2.95	0.56 ± 0.09	0.67 ± 0.29	1.92 ± 0.33	2.63 ± 0.30	2.59 ± 0.33	2.47 ± 1.00
WS3	0.22	0.10 ± 0.08	0.34	0 ± 0.01	0 ± 0.01	0.04 ± 0.03	0.05 ± 0.03	0.03 ± 0.02	0.04 ± 0.04
WS5	0.04	0.10 ± 0.05	0	0 ± 0.01	0 ± 0.01	0.02 ± 0.01	0.04 ± 0.03	0.05 ± 0.02	0.07 ± 0.04
unclassified	1.11	1.07 ± 0.12	1.05	0.50 ± 0.08	0.44 ± 0.08	0.64 ± 0.09	0.76 ± 0.02	1.01 ± 0.04	0.86 ± 0.15

Supplementary table S5. Relationship between incubation time and relative abundance of Archaeal functional gene categories at subsystem level 1 (SEED database). Only classification levels having P < 0.05 were shown.

**Functional classification**	**Regression**		
**Level 1**	**P**	**R^2**	**Coefficient**
Carbohydrates	<0.001	0.654	-0.16548
Cell Wall and Capsule	<0.001	0.583	-0.06881
Cofactors, Vitamins, Prosthetic Groups, Pigments	0.002	0.442	0.04291
Membrane Transport	0.004	0.389	-0.03033
Metabolism of Aromatic Compounds	<0.001	0.534	-0.01349
Motility and Chemotaxis	0.001	0.497	-0.02598
Nucleosides and Nucleotides	<0.001	0.703	0.051226
Phages, Prophages, Transposable elements, Plasmids	0.024	0.265	0.009792
Phosphorus Metabolism	0.011	0.325	-0.01988
Photosynthesis	0.007	0.357	-0.00816
Potassium metabolism	0.001	0.479	-0.0172
Protein Metabolism	<0.001	0.630	0.18041
RNA Metabolism	<0.001	0.807	0.15122
Stress Response	<0.001	0.632	-0.03717
Sulfur Metabolism	<0.001	0.522	0.02048
Virulence, Disease and Defense	<0.001	0.630	-0.027
